# Pulmonary Cryptococcosis During Osimertinib Treatment for Epidermal Growth Factor Receptor (EGFR) L858R-Mutant Lung Adenocarcinoma: A Case Report

**DOI:** 10.7759/cureus.83929

**Published:** 2025-05-11

**Authors:** Yuki Hamada, Toshiaki Motegi, Kenya Kuramoto, Tatsuya Akiyama, Shigen Hayashi

**Affiliations:** 1 Respiratory Medicine, Ibaraki Seinan Medical Center Hospital, Sakai, JPN; 2 Respiratory Medicine, Tsukuba Medical Center Hospital, Tsukuba, JPN

**Keywords:** cryptococcosis, lung adenocarcinoma, osimertinib, serum cryptococcal antigen, β-d-glucan

## Abstract

Pulmonary cryptococcosis is a rare but important opportunistic fungal infection that may occur during epidermal growth factor receptor-tyrosine kinase inhibitor (*EGFR*-TKI) therapy for lung cancer. We describe the case of a woman in her 60s receiving osimertinib for *EGFR* L858R-mutant lung adenocarcinoma who developed new pulmonary lesions. A CT-guided biopsy confirmed pulmonary cryptococcosis despite negative serum cryptococcal antigen results, with markedly elevated serum β-D-glucan levels, an atypical finding in cryptococcal infections. The patient was also receiving dexamethasone for brain metastases. Although her lymphocyte count was within the normal range, functional immunosuppression was suspected due to the combined effects of *EGFR*-TKItherapy and corticosteroid use. This case highlights the limitations of serological testing in immunocompromised patients undergoing molecular-targeted therapy and underscores the importance of early pathological evaluation. The patient responded well to induction therapy with amphotericin B and flucytosine, followed by fluconazole consolidation. While causality cannot be definitively established, this case suggests the need for vigilance for opportunistic infections in similar clinical settings.

## Introduction

Pulmonary cryptococcosis is a deep-seated fungal infection caused by *Cryptococcus neoformans* or *Cryptococcus gattii*, most commonly seen in immunocompromised individuals such as those with human immunodeficiency virus (HIV) infection or organ transplantation [1]. The infection typically affects the lungs but may disseminate to the central nervous system (CNS). The standard treatment depends on the immune status and disease severity: mild-to-moderate pulmonary disease in immunocompetent hosts can be managed with fluconazole monotherapy. At the same time, immunocompromised patients or those with CNS involvement generally require induction therapy with amphotericin B and flucytosine, followed by fluconazole consolidation [2]. Although rare, it may also occur in cancer patients undergoing molecular targeted therapy [3]. Lung adenocarcinoma is the most common subtype of non-small cell lung cancer. In patients harboring epidermal growth factor receptor (*EGFR*) mutations such as L858R or exon 19 deletions, *EGFR*-tyrosine kinase inhibitors (*EGFR*-TKI) have become standard first-line therapy, with osimertinib showing efficacy against both systemic and CNS disease [4]. *EGFR*-TKI, including osimertinib, has been shown to suppress T-cell proliferation and cytokine production, potentially impairing antifungal immunity even when lymphocyte counts are within the normal range [5,6]. We report a case of pulmonary cryptococcosis that developed during *EGFR*-TKI and corticosteroid therapy, highlighting the diagnostic challenges posed by atypical serological findings and the importance of early histopathological evaluation.

## Case presentation

A woman in her 60s was diagnosed in X year Y−3 month with primary adenocarcinoma of the right upper lobe of the lung harboring an *EGFR* L858R mutation, along with multiple pulmonary and brain metastases. Treatment with osimertinib (80 mg/day, orally) was initiated immediately after diagnosis. Whole-brain radiotherapy was administered for the management of brain metastases. Tumor shrinkage was observed on follow-up imaging performed in Y−2 and Y−1 months. Dexamethasone was initiated at 3.3 mg/day in Y−1 month to alleviate peritumoral edema. As cerebral edema worsened later that month, the dose was increased to 13.2 mg/day. By admission, it had been gradually tapered to 4 mg/day.

**Figure 1:**
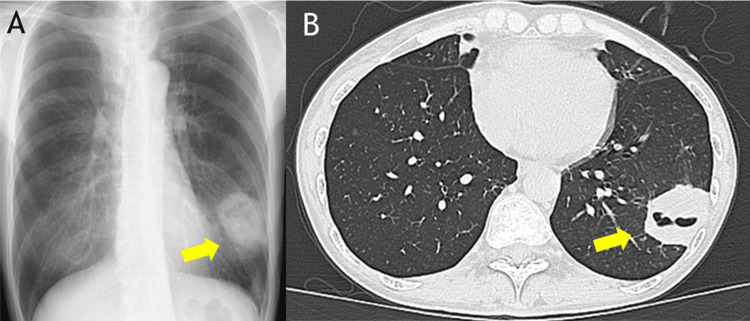
Imaging findings on admission (X year Y month) (A) Chest X-ray showing a newly appeared mass shadow in the left lower lung field (yellow arrow). (B) Chest CT scan showing a newly developed cavitary lesion with wall thickening in the left lower lobe (yellow arrow). CT: computed tomography

In Y month, during routine follow-up, chest CT revealed a newly developed cavitary mass with wall thickening in the left lower lobe and multiple nodular shadows in the left upper lobe, raising clinical suspicion for disease progression (Figure 1). The patient was admitted for further evaluation.

On admission, she was asymptomatic, with no complaints of cough, sputum production, fever, or chest pain. She denied any recent travel or contact with birds, including pigeons, and had no smoking history. She was alert and oriented. Vital signs were within normal limits: blood pressure 116/64 mmHg, pulse rate 67 bpm, and temperature 36.7°C. Chest auscultation and physical examination revealed no abnormalities.

Laboratory investigations revealed an elevated carcinoembryonic antigen (CEA) level and a mildly increased C-reactive protein (CRP). The white blood cell count and lymphocyte percentage were decreased, and the absolute lymphocyte count was markedly reduced compared to the institutional lower limit, suggesting a possible state of cellular immunosuppression (Table 1).

**Table 1:** Laboratory investigations and changes in tumor and fungal markers during hospitalization Both CEA and β-D-glucan levels showed a significant decline following antifungal therapy. WBC: white blood cells, CRP: C-reactive protein, CEA: carcinoembryonic antigen, β-D-glucan: beta-D-glucan

Test	On admission	Hospital day 55	Reference range
WBC (/μL)	3,600	-	4,400-11,000
Lymphocytes (%)	12.4	-	20-50
Absolute lymphocyte count	446	-	1,000-4,800
CRP (mg/dL)	0.26	-	<0.3
CEA (ng/mL)	8.15	5.1	<5.0
β-D-glucan (pg/mL)	390	10.1	<20

**Figure 2:**
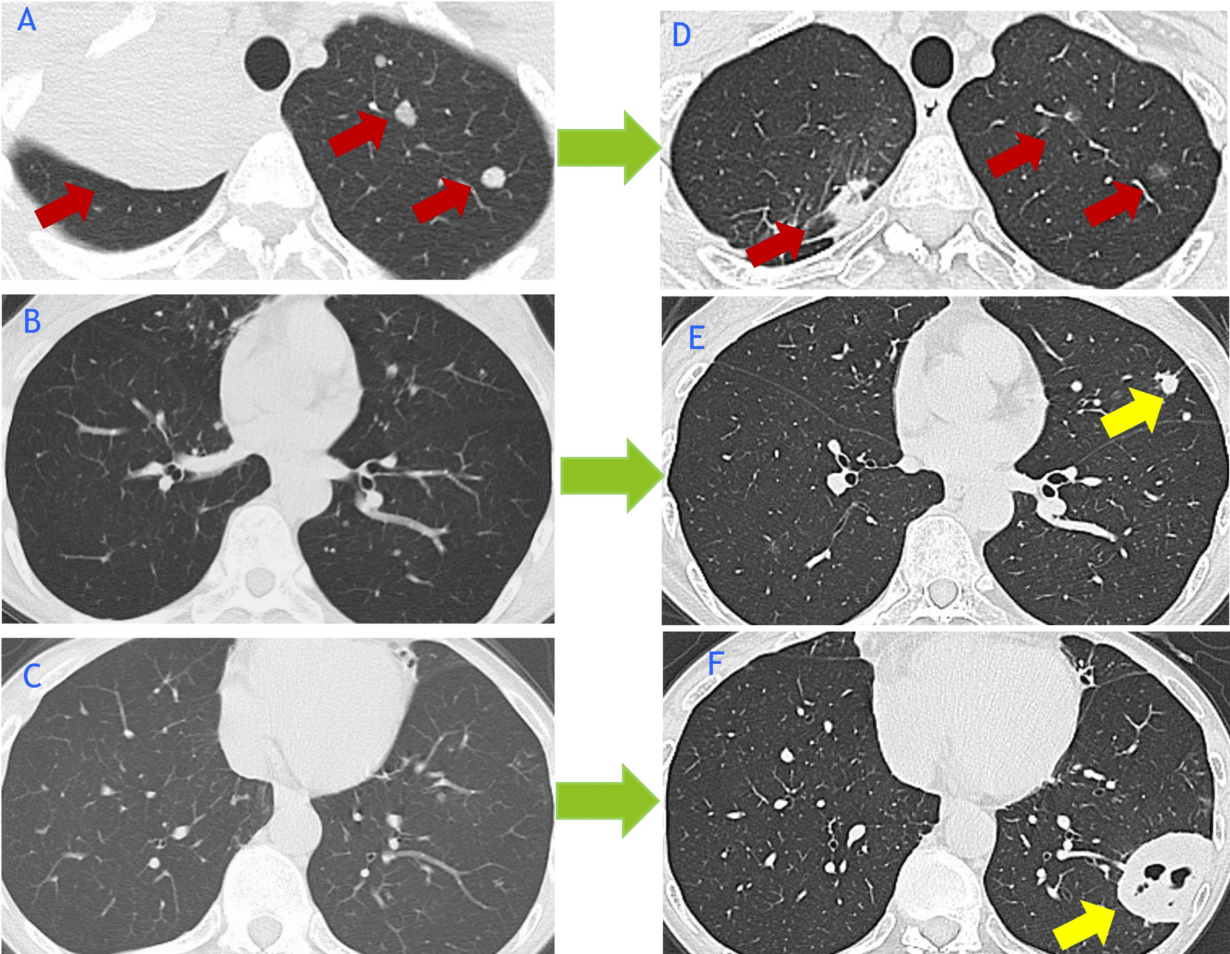
Chest CT findings before and after osimertinib treatment Chest CT images were obtained in X year Y-3 month (A–C) and Y month (D–F). Red arrows indicate tumor lesions that decreased in size following treatment with osimertinib. Yellow arrows indicate newly developed infiltrative shadows and cavitary lesions, suggestive of either disease progression or secondary infection. CT: computed tomography

Chest radiography revealed a new mass in the left lower lung field. CT confirmed shrinkage of the right upper lobe primary lesion and the known pulmonary metastases, but also demonstrated new multiple nodules in the left upper lobe and a cavitary mass in the left lower lobe (Figures 1-2).

**Figure 3:**
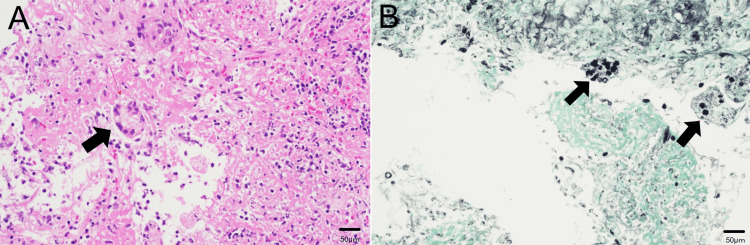
Histopathological findings from CT-guided lung biopsy of the left lower lobe (A) H&E staining showing multinucleated giant cells containing round, unstained, haloed structures (arrowheads). (B) Grocott’s methenamine silver stain highlighting spherical fungal organisms morphologically consistent with *Cryptococcus *species (arrows). These findings supported a presumptive diagnosis of secondary pulmonary cryptococcosis in an immunocompromised patient. CT: computed tomography, H&E: hematoxylin and eosin

Given that *EGFR*-mutant adenocarcinoma can exhibit rapid progression, a CT-guided percutaneous lung biopsy was performed on the left lower lobe mass on hospital day 2 to exclude malignancy. Histopathological examination revealed no evidence of malignancy. Hematoxylin and eosin (H&E) staining showed multinucleated giant cells containing round, unstained, haloed structures, while Grocott's methenamine silver staining demonstrated fungal organisms morphologically consistent with *Cryptococcus* species (Figure 3). Although culture and molecular identification were not performed or were inconclusive, the morphological findings were considered highly suggestive of cryptococcal infection. In the context of a known immunocompromised state due to cancer and corticosteroid therapy, these findings supported a presumptive diagnosis of secondary pulmonary cryptococcosis.

Serologic testing revealed a negative serum cryptococcal antigen (CrAg) result, while β-D-glucan was markedly elevated (Table 1). Although a prozone effect was a possible explanation for the negative CrAg result, it was not recognized at the time of testing, and therefore, repeat testing using diluted serum was not performed. No CrAg testing or polymerase chain reaction (PCR) was performed on the biopsy specimen due to institutional constraints and lack of insurance coverage in Japan. The Japanese healthcare insurance system does not routinely cover these tests, limiting their accessibility. *Cryptococcus* antigen was measured using the Serodirect Eiken *Cryptococcus* kit (Eiken Chemical Co., Ltd., Tokyo, Japan) by latex agglutination. The reaction was performed using the TESMIC rotator and KTH-60TS thermostatic water bath (both from System Stage Tokyo Co., Ltd., Tokyo, Japan) and β-D-glucan was measured using the Fungitec G Test MK II (Shimada Diagnostic Co., Ltd., Tokyo, Japan) based on a colorimetric synthetic substrate method, with the WELL Reader SK603 (Scinics Inc., Tokyo, Japan).

Due to concern for CNS dissemination, a lumbar puncture was performed. Cerebrospinal fluid (CSF) analysis revealed no abnormalities, and CSF CrAg was also negative, excluding cryptococcal meningitis.

Empiric antifungal therapy was initiated with intravenous liposomal amphotericin B (4 mg/kg/day, 140 mg/day) plus oral flucytosine (100 mg/kg/day, 4000 mg/day in divided doses) on hospital day 10. This combination was continued for 20 days, until hospital day 30. After CNS involvement was ruled out, therapy was de-escalated to oral fluconazole (400 mg/day) beginning on hospital day 30. Dexamethasone was continued during hospitalization to manage peritumoral edema; the dose was gradually tapered and discontinued by the time of discharge. The patient showed a favorable clinical and serological response, with decreased serum CEA and β-D-glucan levels by hospital day 55 (Table 1).

**Figure 4:**
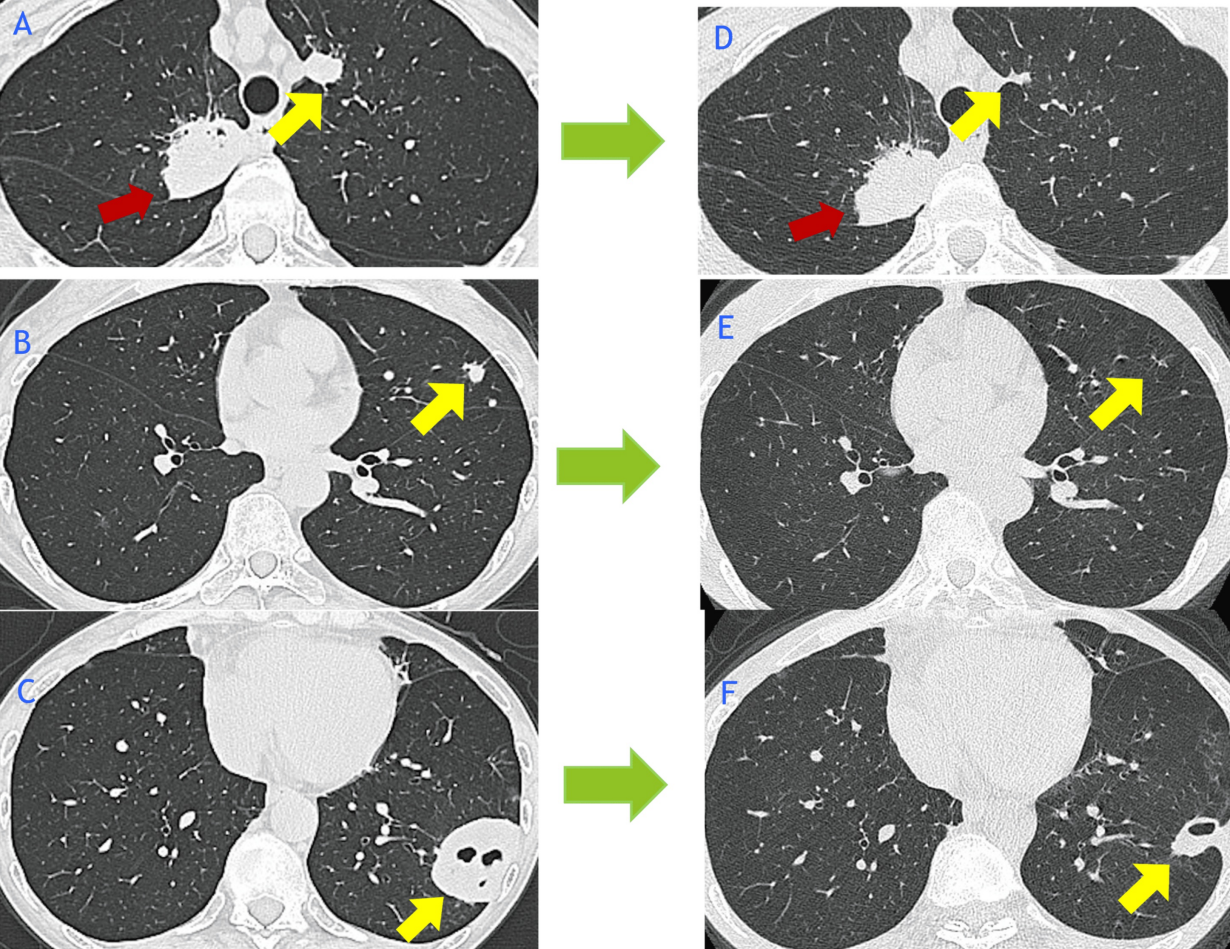
Chest CT findings before and after antifungal therapy for pulmonary cryptococcosis CT images were obtained in X year Y month (A–C) and Y+4 month (D–F). Red arrows indicate the primary lung tumor, which remained stable after treatment. Yellow arrows indicate cryptococcal lesions, which resolved or decreased in size following antifungal therapy. The initial treatment consisted of intravenous liposomal amphotericin B (4 mg/kg/day, 140 mg/day) plus oral flucytosine (100 mg/kg/day, 4000 mg/day in divided doses), administered for 20 days from hospital day 10 to day 30. After CNS involvement was ruled out, the regimen was de-escalated to oral fluconazole (400 mg/day), which was continued thereafter. CT: computed tomography, CNS: central nervous system

The patient was discharged on hospital day 60. Follow-up chest radiography in Y+4 months showed that the cavitary lesion in the left lower lobe had regressed and transformed into a thin-walled cavity (Figure 4).

## Discussion

Pulmonary cryptococcosis presents diverse radiologic features, including nodules, masses, and cavitary lesions, often mimicking malignancy and making differentiation challenging [3]. In the present case, early consideration of an infectious etiology for the newly emerging lesions during treatment and prompt implementation of CT-guided biopsy led to a definitive diagnosis.

Although the CrAg test is a highly sensitive and non-invasive diagnostic tool, negative results may occur in cases of localized pulmonary cryptococcosis. In our case, despite the presence of multiple pulmonary lesions and markedly elevated serum β-D-glucan, the CrAg was negative. A prozone effect was a possible explanation for this result, but it was not recognized at the time of testing; therefore, repeat testing with diluted serum was not performed. This represents an important limitation of the case. Although mucicarmine staining was considered, it was not performed due to the unavailability of reagents at our institution. However, Grocott’s methenamine silver staining revealed yeast-like organisms consistent with *Cryptococcus* species.

Microbiological cultures were negative, and neither PCR nor antigen testing was performed on the tissue specimen due to institutional limitations and lack of insurance coverage in Japan. PCR testing was also not pursued because the expected turnaround time was approximately one month, and clinical decision-making prioritized prompt treatment initiation. The negative culture results may have reflected a low fungal burden or technical limitations in sample acquisition or handling. Although species-level identification was not feasible, *Cryptococcus neoformans* is generally more prevalent in immunocompromised patients, including those undergoing cancer therapy.

While elevated β-D-glucan is uncommon in cryptococcal infections, high levels have been reported in cryptococcal meningitis [7]. In the present case, the β-D-glucan elevation may reflect local fungal burden, fungal lysis, accumulation of dead organisms, or inflammation-induced translocation of β-D-glucan into the bloodstream. Although rare, several reports, particularly in CNS infections, have demonstrated similar β-D-glucan elevation patterns, suggesting that caution is warranted when interpreting such findings.

Co-infection with *Aspergillus* species was also considered, as mixed infections with *Cryptococcus* and *Aspergillus* have been reported in immunocompromised hosts [8]. However, no radiological findings suggest angioinvasive aspergillosis, and no hyphal elements were detected histologically. Furthermore, both *Aspergillus* antigen and antibody tests were negative, making co-infection unlikely.

Other potential causes of cavitary lung lesions, such as tuberculosis, nocardiosis, and endemic mycoses, were also evaluated. Acid-fast bacillus smears, *Mycobacterium tuberculosis* PCR, and mycobacterial cultures were all negative. The patient had no epidemiologic risk factors for endemic fungal infections. Additionally, an HIV antibody test was negative, ruling out HIV-associated immunodeficiency, which is standard practice in evaluating cryptococcal infections.

*EGFR* is expressed not only in tumor cells but also in CD4+ T lymphocytes. *EGFR*-TKI has been reported to impair T-cell proliferation and reduce cytokine production, including interferon-γ (IFN-γ), interleukin-2 (IL-2), and interleukin-4 (IL-4), which are essential for antifungal defense [5,6]. IFN-γ, in particular, plays a central role in macrophage activation for cryptococcal clearance. In this case, the absolute lymphocyte count was approximately 450/μL, indicating mild lymphopenia. Although not profound, this reduction may reflect impaired cell-mediated immunity. Functional suppression of Th1-type responses due to *EGFR*-TKI therapy could have contributed to the development of cryptococcosis.

Corticosteroids such as dexamethasone are also well known to suppress cell-mediated immunity and increase susceptibility to opportunistic fungal infections, including cryptococcosis and aspergillosis [9,10]. Specifically, dexamethasone has been shown to impair human macrophages' antifungal activity and promote the growth of *Aspergillus fumigatus* in vitro [10]. These findings suggest that corticosteroid use may independently contribute to impaired host defense and should be considered a significant confounding factor when evaluating the immunosuppressive impact of *EGFR*-TKI therapy.

In this case, the possibility of a prozone phenomenon [11] and the involvement of atypical serotypes such as *Cryptococcus gattii*, which has lower CrAg detection sensitivity [12], cannot be excluded. Additionally, capsule-deficient strains, which are known to exhibit reduced CrAg detectability [13], may also have played a role.

A previous case of pulmonary cryptococcosis during EGFR-TKI therapy was reported by Kobe et al. [14]. However, to our knowledge, no prior reports have described a CrAg-negative and β-D-glucan-positive serologic profile. Our case thus represents a rare and atypical presentation of pulmonary cryptococcosis occurring during molecular targeted therapy. It was characterized by multiple unusual features: CrAg-negative/β-D-glucan-positive serology, imaging findings indistinguishable from tumor progression, and preserved lymphocyte counts despite apparent functional immunosuppression. This case underscores the need for a multifaceted immune assessment and infection surveillance approach in patients receiving targeted therapies.

This case report has several limitations. First, the diagnosis was based on histopathological findings without species-level identification or molecular confirmation. Second, as diluted CrAg testing was not performed due to a lack of recognition of the prozone phenomenon, its contribution cannot be completely ruled out retrospectively. Third, using corticosteroids represents a confounding factor that complicates the interpretation of the immunosuppressive role of *EGFR*-TKI therapy. Finally, as a single case report, the generalizability of our observations is inherently limited and should be interpreted cautiously.

## Conclusions

When new pulmonary lesions arise during lung cancer treatment, clinicians must consider infectious etiologies in addition to tumor progression. Even in the setting of negative serologic testing, a comprehensive diagnostic approach, including histopathologic evaluation, is essential. CT-guided percutaneous biopsy is a rapid and minimally invasive diagnostic tool and should be considered in selected cases where diagnostic uncertainty remains. This case suggests that typical laboratory parameters may not reliably reflect immune competence in patients receiving targeted therapies. A proactive diagnostic strategy, including early imaging review and timely tissue sampling, may help ensure accurate diagnosis and avoid misinterpretation of tumor progression. As this is a single case report, the findings should be interpreted with caution.
